# Complete Genome Sequencing of Occult Hepatitis B Virus in Hemodialysis Patients Reveals Subgenotype D2 and Immune Escape Mutations in Bangladesh

**DOI:** 10.1155/ijm/9533013

**Published:** 2026-09-07

**Authors:** Roni Mia, Anandha Mozumder, Parsa Irin Disha, Nashid Sultana Ishi, S. M. Nazmul Hasan, Anupam Das, Chirojit Debnath, M. Shaminur Rahman, Sharmin Akter, S. M. Rashed Ul Islam, Sukumar Saha, Tofazzal Islam, Muzahed Uddin Ahmed, Chitta Ranjan Debnath, Md. Golzar Hossain

**Affiliations:** ^1^ Department of Microbiology and Hygiene, Bangladesh Agricultural University, Mymensingh, Bangladesh, bau.edu.bd; ^2^ Department of Microbiology, Mymensingh Medical College, Mymensingh, Bangladesh, mmc.gov.bd; ^3^ Department of Hepatology, Mymensingh Medical College, Mymensingh, Bangladesh, mmc.gov.bd; ^4^ Department of Microbiology, Jashore University of Science and Technology, Jashore, Bangladesh, just.edu.bd; ^5^ Department of Physiology, Bangladesh Agricultural University, Mymensingh, Bangladesh, bau.edu.bd; ^6^ Department of Virology, Bangladesh Medical University, Dhaka, Bangladesh; ^7^ Institute of Biotechnology and Genetic Engineering, Gazipur Agricultural University, Gazipur, Bangladesh; ^8^ Department of Medicine, Bangladesh Agricultural University, Mymensingh, Bangladesh, bau.edu.bd

**Keywords:** HBV genome sequencing, hemodialysis patients, mutational analysis, occult Hepatitis B infection (OBI), phylogeny

## Abstract

Hepatitis B virus (HBV) remains a major global health concern, and occult HBV infection (OBI) presents significant diagnostic and clinical challenges, particularly among hemodialysis (HD) patients. This study is aimed at characterizing complete HBV genomes from maintenance HD patients with OBI in Bangladesh to elucidate genetic features, mutational patterns, and clinical implications. Serum samples from two HBsAg‐negative HD patients were screened by ELISA and quantitative PCR. Viral DNA was amplified by PCR across four overlapping open reading frames (ORFs) and sequenced on the Illumina platform. Genome assembly, phylogenetic analysis, and mutational profiling were performed using reference datasets and bioinformatics tools. Antigenicity and hydrophilicity of HBsAg were predicted in silico. Both patients were anti‐HBc and anti‐HBs positive with high HBV DNA loads (2.29 × 10^10^ and 2.53 × 10^10^ copies/mL). Full‐length genomes (3182 bp) were successfully sequenced and phylogenetic analysis showed both HBV genomes clustered within Genotype D, Subgenotype D2, and subtype ayw3, consistent with previously reported Bangladeshi HBV genomes. Comparative mutational analysis identified substitutions such as T1753C in the basal core promoter, C1845T in preC, and D144E within the “a” determinant of HBsAg, suggesting potential roles in vaccine escape, immune escape, and diagnostic failure. Several nonsynonymous mutations were also detected in polymerase, though none were potentially associated with antiviral resistance. Antigenicity and hydrophilicity profiles of HBsAg and its major hydrophilic region remained largely conserved. These findings demonstrate the persistence of OBI in HD patients and provide an initial indication of the need for genomic surveillance to monitor immune‐escape mutations and improve HBV diagnostic strategies in endemic regions.

## 1. Introduction

Hepatitis B virus (HBV) remains a major global public health concern. An estimated 2.57 billion people have been infected worldwide, and approximately 296 million suffer from chronic infection, placing them at increased risk of HBV‐associated complications such as liver cirrhosis (LC) and hepatocellular carcinoma (HCC) [1–3].

HBV infection is typically identified by the presence of Hepatitis B surface antigen (HBsAg) and detectable virus in blood. However, in the late 1970s, a “silent” or “occult” form of HBV was discovered among blood donors who tested negative for HBsAg but were anti‐HBc positive and still capable of transmitting the virus [4]. This condition, now recognized as occult Hepatitis B infection (OBI), has remained a subject of debate for over 4 decades regarding its clinical significance [5]. OBI is defined as the presence of HBV DNA in the liver or blood of individuals who test negative for HBsAg with currently available assays [6]. Based on HBV serological markers, OBI can be classified as either seropositive (anti‐HBs and/or anti‐HBc positive) or seronegative (anti‐HBs and anti‐HBc negative) [7]. OBI is widespread globally, including in Bangladesh, where HBV infection is highly endemic [8].

The persistence of OBI is often linked to mutations in the small HBsAg gene, resulting in defective or altered surface proteins that evade detection by standard assays, even when viral DNA levels are comparable with HBsAg‐positive cases [9–11]. The “a” determinant within the major hydrophilic region (MHR) of HBsAg is a key antigenic domain critical for inducing protective immune responses [10, 12, 13]. Mutations in this region can facilitate immune evasion, reduce vaccine effectiveness, and lead to false‐negative HBsAg results, thereby sustaining occult infection [9, 13, 14]. In addition, long‐term persistence of covalently closed circular DNA (cccDNA) within hepatocyte nuclei plays a central role in maintaining OBI [15].

OBI has important clinical implications, particularly in patients with chronic kidney disease (CKD) undergoing hemodialysis (HD). These patients are highly vulnerable to HBV because of repeated vascular access and impaired immunity [16, 17]. Globally, the prevalence of HBV infection in CKD patients ranges from 2% to over 20%, with higher rates in regions of intermediate to high endemicity, including Bangladesh [18, 19]. HBV infection in CKD patients is associated with accelerated liver disease progression, increased risk of cirrhosis and HCC, and poor vaccine responsiveness [20, 21]. Furthermore, HBV poses a serious risk of nosocomial transmission in dialysis units, complicating patient management and long‐term outcomes [22]. Reactivation of HBV following renal transplantation can cause acute hepatitis, progressive liver disease, graft loss, and even fatal outcomes [16, 23].

In Bangladesh, the majority of OBI cases go undetected because predialysis screening typically relies solely on HBsAg testing [8]. Although some studies have incorporated anti‐HBc testing to improve detection, these efforts highlight the urgent need for more comprehensive diagnostic strategies [24, 25]. Accurate detection of OBI is essential to minimize transmission risk and improve patient outcomes [26]. Because HBV mutations are central to OBI pathogenesis, genomic characterization of viral strains in HD patients is critical [9, 11]. However, complete genome sequencing and mutational analysis of HBV in Bangladeshi CKD and HD patients with OBI remain scarce. This lack of genome‐wide data limits understanding of clinically relevant mutations related to immune escape, diagnostic failure, and antiviral resistance, underscoring the importance of genomic surveillance in this high‐risk group.

Therefore, the present study was designed to sequence the complete HBV genome from HD patients with OBI using a modified PCR‐based amplification of four ORFs combined with Illumina sequencing technology. In addition, the study aims to investigate phylogenetic relationships and nucleotide polymorphisms potentially associated with OBI, immune escape, and drug resistance.

## 2. Methods and Materials

### 2.1. Ethical Approval

The study followed ethical guidelines and obtained approval from the Institutional Review Board (IRB) under the reference number MMC/IRB/2024/678. Patients participating in this investigation provided their consent in written form and verbally.

### 2.2. Sample Collection and Preparation

**Figure 1:**
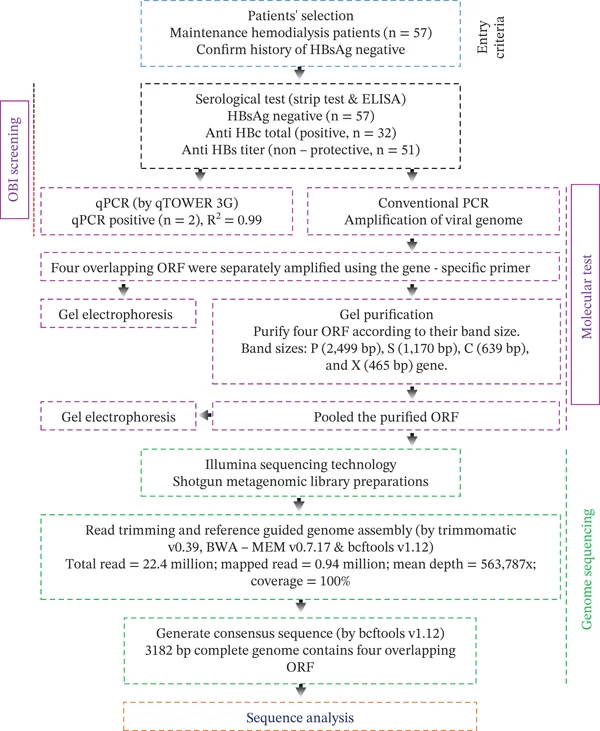
Study workflow. Schematic representation of the study workflow illustrating the sequential steps of the research process. The diagram was created using Microsoft PowerPoint.

A total of 57 patients were selected receiving maintenance hemodialysis (MHD) and had a confirmed history of being HBsAg‐negative (Figure 1). Approximately 4 mL of blood was carefully collected and placed into a red‐capped tube (Boen Healthcare Co. Ltd., China) under aseptic procedure from the dialysis catheter before its connection to the dialysis machine. All patients were receiving treatment at the Department of Nephrology, Mymensingh Medical College Hospital, Mymensingh, Bangladesh. The samples were then transported to the Laboratory of Virology, Department of Microbiology and Hygiene at Bangladesh Agricultural University, within 30 min after collection. Following clot formation, the serum was separated from the clotted blood and centrifuged at 3000 rpm for 10 min at room temperature to eliminate any remaining blood clot. The separated serum was then collected and aliquoted into 1.5 mL microcentrifuge tubes and stored at −20°C until analysis.

### 2.3. Immunochromatographic Test (ICT)

The HBsAg detection was carried out using the Active Quick Test Strip (ISO 13485:2016 certified), Hangzhou AllTest Biotech Co. Ltd., China. Two to three drops of serum were added to the test well, and the results were interpreted by noting the color changes as per the manufacturer′s guidelines.

### 2.4. Enzyme‐Linked Immunosorbent Assay (ELISA)

Three commercially available ELISA kits: anti‐Hepatitis B Surface Antigen (quantitative) ELISA, Hepatitis B Surface Antigen (HBsAg) ELISA, and Antobody to Hepatitis B Core Antigen (HBc Ab) ELISA (all from Biogen GmbH, Prenzlauer Promendae 147, D‐13189 Berlin, Germany) were used. Preparation of reagents, including control/sample diluents, wash buffers, and substrate solutions, followed the manufacturer′s instructions. The assays were carried out strictly according to the protocols provided with each kit. Optical density was read at 450 nm using a Multiskan FC microplate reader (Ref.: 51119000, Thermo Scientific, Singapore). Result interpretation and cutoff value were determined based on the control parameters provided with the kits.

### 2.5. DNA Extraction

The viral DNA was extracted from the serum using the TIANamp Virus DNA/RNA Kit (Cat: DP304‐03, Tiangen, China), following the manufacturer′s protocol. The extracted DNA was used for quantitative polymerase chain reaction (qPCR) and to prepare the sequencing template. The qPCR was performed on a qTOWER 3G qPCR machine (Analytik Jena, Germany) using HRbio qPCR SYBR Green Master Mix (High Rox Plus) (Cat: HRF0052, HeruiBio, China). Each 20 *μ*L qPCR reaction contained the specific qPCR primers, HBs F2 (5 ^′^‐CTTCATCCTGCTGCTATGCCT‐3 ^′^) and HBs R2 (5 ^′^‐AAAGCCCAGGATGATGGGAT‐3 ^′^). The thermal profile included an initial denaturation at 50°C for 2 min, followed by 95°C for 2 min; then 40 amplification cycles of 95°C for 1 s and 60°C for 30 s; ending with a melting curve analysis from 55°C to 95°C over 15 s. All assays were run in triplicate and mean of viral loads was determined based on a standard curve.

### 2.6. PCR Amplification of Complete HBV ORFs

Conventional PCR was performed using four pair of primer sets (Table 1) to amplify four complete HBV ORF, P (2499 bp), S (1170 bp), C (639 bp), and X (465 bp). PCR reaction (50 *μ*L) contained 2.5 *μ*L (0.5 *μ*M concentration) of each gene‐specific primer (forward and reverse), 17.5 *μ*L DNA template, 25 *μ*L Master Mix (TaKaRa Taq Version 2.0 plus dye, Takara, Cat: RR901A Japan), and 2.5 *μ*L double‐distilled water (DDW). The amplification was carried out under the following cycling conditions: an initial denaturation at 94°C for 5 min, followed by 35 cycles consisting of denaturation at 94°C for 30 s, annealing at 54°C for 30 s, and extension at 60°C for 2 min. A final extension was performed at 60°C for 7 min. Amplified products were then subjected to electrophoresis on a 1.5% agarose gel and stained with ethidium bromide. The electrophoresis was conducted at 100 V for 30 min and visualized using a UV illuminator (ChemiDoc Go Imaging System, Bio‐Rad, United States).

**Table 1:** List of primers used in this study.

Primer	Sequence (5 ^′^‐3 ^′^)	Region	Amplicon size (bp)	Positions	Reference
EcoRI‐Pol‐1	GTGGAATTCGGATGCCCCTATCTTATCAACAC	P	2499	2307–3182, 1–1623	[27]
Pol‐stop‐SalI	CACGTCGACTCACGGTGGTCTCCATGCGAC				
EcoRI‐HBs preS1	GTGGAATTCGGATGGGAGGTTGGTCTTCCAAAC	S	1170	2848–3182, 1–835	
HBs‐stop‐SalI	CACGTCGACTTAAATGTATACCCAAAGAC				
EcoRI‐HBx‐1	GTGGAATTCGGATGGCTGCTAGGGTGTGCTG	X	465	1374–1838	
HBx‐stop‐SalI	CACGTCGACTTAGGCAGAGGTGAAAAAGTTG				
EcoRI‐Core‐1	GTGGAATTCGGATGGACATTGACCCGTATAAAG	C	639	1814–2452	
Core‐stop‐SalI	CACGTCGACTAACATTGAGATTCCCGAG				
HBs F2	CTTCATCCTGCTGCTATGCCT	qPCR	222	406–627	
HBsR2	AAAGCCCAGGATGATGGGAT				

### 2.7. Gel Purification

DNA fragments of different HBV ORF were excised from the agarose gel under a UV illuminator. Four fragments were purified separately using a commercial Gel Extraction Kit (Cat: HRQ0633, HeruiBio, China). The purified products were pooled and subjected to whole‐genome sequencing as template.

### 2.8. Sequencing and Genome Assembly

Complete HBV viral genome was sequenced using Illumina sequencing technology (Illumina NovaSeq 6000, United States). Shotgun metagenomic libraries were generated following a modified iNextEra workflow. DNA tagmentation was carried out using Illumina bead‐linked transposomes (Illumina DNA Library Prep, Illumina Inc., United States). The resulting libraries were pooled and sequenced on the Illumina NovaSeq X Plus system (2 × 150 bp; Novogene, United States). Raw paired‐end reads underwent adapter removal and quality trimming with Trimmomatic v0.39, keeping sequences longer than 50 bp. Data quality was evaluated using FastQC v0.11.9. Filtered reads were mapped to the HBV reference genome (GenBank accession NC_003977.2; 3,182 bp) via BWA‐MEM v0.7.17 with default settings [28]. Alignment files were subsequently processed with Samtools v1.12 [29], variant calling was performed using FreeBayes v1.3.5 [30], and a consensus genome sequence was constructed using bcftools v1.12 [31]. For confirmation of the complete genome of our identified HBV, the sequences were BLAST searched against the NCBI database and aligned with the reference genome. Following that, the sequences were annotated using CLC sequence viewer (Version 8.0). Identified HBV were designated as MGH_HBV‐076 and MGH_HBV‐104.

### 2.9. Phylogenetic Analysis

A total of 97 HBV complete genome sequences of different genotypes and their subgenotypes were retrieved in FASTA format from the NCBI GenBank database. Additionally, 96 HBV complete genomes of different subgenotypes under only D genotype were also retrieved from the NCBI. Multiple sequence alignment of collected sequences and identified two HBV (MGH_HBV‐076 and MGH_HBV‐104) was performed using the MUSCLE program (v5.2) [32], and a phylogenetic tree was constructed by using the IQ‐TREE 2 (v2.1) program [33] with 1000 bootstrap replicates on the Linux operating system, and the tree was visualized under iTOL (https://itol.embl.de/) online server.

### 2.10. Mutational Analysis

A recent well‐characterized complete HBV genome sequence originating from Bangladesh used as a refences retrieved from NCBI (GenBank accession no. MF925358.1). The complete genome sequences of MGH_HBV‐076 and MGH_HBV‐104 were aligned with the reference genome to identify nucleotide substitutions. To determine amino acid (aa) changes, the four overlapping genes (P, S, X, and C) of both identified HBV genomes and the reference sequence were translated and aligned using CLC Sequence Viewer (Version 8.0). HBV drug resistance, vaccine, diagnostic, and immune escape mutations analyses were performed according to the previously published report [34] and geno2pheno (https://hbv.geno2pheno.org/) online tool.

### 2.11. Antigenicity and Hydrophilicity Analysis

Antigenicity and hydrophilicity profile were determined by using the IEDB online tools (https://www.iedb.org/) under the Kolaskar and Tongaonkar antigenicity (a linear sequence‐based prediction approach) and Parker hydrophilicity prediction parameter, respectively. The HBsAg MHR and “a” determinant protein region were submitted to the server for their antigenicity and hydrophilicity profile under the default settings. The generated figures and data were retrieved analyzed.

### 2.12. Statistical Analysis

The chi‐square test was performed to assess the distribution of categorical variables related to sociodemographic and clinical characteristics among patients undergoing MHD. A *p*‐value of < 0.05 was considered statistically significant. All analyses were conducted using Microsoft Excel and R program.

## 3. Results

### 3.1. Sociodemographic and Clinical Characteristics of the MHD Patients

The mean age of the participants was 51.35 ± 14.59 years, with the majority aged ≥ 60 years (36.86%), followed by 50–59 years (24.56%), 30–39 years (19.29%), 40–49 years (12.28%), and ≤ 29 years (7.01%) with significant difference (*p* = 0.0043) (Table 2). Males constituted the majority (70.18%) compared with females (29.82%) (*p* = 0.002). Occupational distribution varied, with housewives and retired individuals each comprising 21.05%, followed by businessmen (15.79%), farmers (12.28%), unemployed (12.28%), and other occupations (17.54%) with *p* = 0.748. A family history of HBV infection was reported in 7.01% of patients (*p* < 0.001) (Table 2). Most patients received twice‐weekly dialysis (85.96%) (*p* < 0.001), and 71.93% had AVF established prior to MHD initiation. Blood transfusion history was present in 85.96% of patients (Table 3). MHD was initiated electively in 66.67% and emergently in 33.33% (*p* = 0.011). Hypertension was the leading cause of CKD (89.47%), followed by diabetes mellitus (29.82%); in both cases males are predominant. The majority were unvaccinated (85.96%) (*p* < 0.001) (Table 3).

**Table 2:** Sociodemographic characteristics of study populations. Information was collected by using a predesigned questionnaire.

Sociodemographic characteristics		Number of patients *n* (%)	*p*
Age	Average ± SD	51.35 ± 14.59	
	≤ 29	4 (7.01)	0.0043^S^
	30–39	11 (19.29)	
	40–49	7 (12.28)	
	50–59	14 (24.56)	
	≥ 60	21 (36.86)	

Sex	Female	17 (29.82)	0.002^S^
	Male	40 (70.18)	

Occupations	Businessman	9 (15.79)	0.748^NS^
	Farmer	7 (12.28)	
	Housewife	12 (21.05)	
	Retired from service	12 (21.05)	
	Unemployed	7 (12.28)	
	Other occupations	10 (17.54)	

Family history of HBV	Yes	4 (7.018)	< 0.001^S^
	No	53 (92.98)	

*Note: p* value was determined by chi‐square test using R program.

Abbreviations: NS, nonsignificant; S, significant; SD, standard deviation.

**Table 3:** Clinical characteristics and serological profile of study populations. Information was collected by using a predesigned questionnaire.

Clinical characteristics		Number of patients *n* (%)	*p*
Number of dialysis (per week)	One	8 (14.04)	< 0.001^S^
	Two	49 (85.96)	
AVF was made prior MHD		41 (71.93)	
Blood transfusion history		49 (85.96)	
Initiation of MHD	Elective	38 (66.67)	0.011^S^
	Emergency	19 (33.33)	
Causes of CKD	DM	17 (29.82)	
	HTN	51 (89.47)	
Vaccinations	No	49 (85.96)	< 0.001^S^
	Yes	8 (14.04)	
Serological test	HBsAg (negative)	57 (100)	
	Anti‐HBc total (positive)	32 (56.14)	
	Anti‐HBs titer		
	Nonprotective	51 (89.47)	< 0.001^S^
	Protective	6 (10.53)	

*Note:* *p* value was determined by chi‐square test using R program.

Abbreviations: AVF, arteriovenous fistula; CKD, chronic kidney disease; DM, diabetes mellitus; HTN, hypertension; MHD, maintenance hemodialysis; NS, nonsignificant; S, significant.

### 3.2. Serological Evidence of All MHD Patients

A total of 57 patients were evaluated; all patients were HBsAg negative, whereas 56.14% were positive for anti‐HBc total (Table 3). Most patients (89.47%) had nonprotective anti‐HBs levels, with only 10.53% showing protective immunity (*p* < 0.001) based on CDC guidelines, whereas anti‐HBs titers were categorized as nonprotective (< 10 mIU/mL) or protective (≥ 10 mIU/mL).

### 3.3. Serological and Molecular Evidence of OBI in MHD Patients

OBI is characterized by the presence of HBV DNA in hepatic tissue and/or peripheral blood, despite testing negative for HBsAg by conventional assays. In this study, the rapid strip test and ELISA showed that all (57) serum samples from MHD patients were negative for HBsAg. Interestingly, qPCR revealed only that two patients (3.50%) had high viral copy number in serum with standard curve values of *R* ^2^ = 0.99 and viral load 2.29 × 10^10^ and 2.53 × 10^10^ copies/mL, respectively (Table 4). Additionally, both patients tested positive for total anti‐HBc and anti‐HBs, although the anti‐HBs titers remained below the protective threshold. These findings indicate that the MHD patients were suffering from OBI. Both patients also had a clinical history of multiple blood transfusions and hypertension (Table 4).

**Table 4:** OBI patient information. Data were gathered using a predesigned questionnaire.

Parameters		Patients ID	
		ID‐076	ID‐104
Date of diagnosis		2019	2019
Number of dialysis		2/wk	1/wk
AVF was made MHD		Yes	No
Multiple blood transfusion		Yes	Yes
Initiation of MHD		Elective	Emergency
Causes of CKD	DM	No	No
	HTN	Yes	Yes
	CGN	No	No
	ADPKD	No	No
Vaccine		No	No
ELISA	HBsAg	Negative	Negative
	Value	0.7877	0.2185
	Anti‐HBc total	Positive	Positive
	Value	0.0666	0.434
	Anti‐HBsAg	Nonprotective	Nonprotective
	Value	0.1537	0.0541
qPCR	Ct value	12.3	10.79
	Copy number	2.29 × 10^10^ copies/mL	2.53 × 10^10^ copies/mL

Abbreviations: ADPKD, autosomal dominant polycystic kidney disease; AVF, arteriovenous fistula; CKD, chronic kidney disease; CGN, chronic glomerulonephritis; DM, diabetes mellitus; HTN, hypertension; MHD, maintenance hemodialysis.

### 3.4. Amplification and Assembly of Full‐Length HBV Genomes

**Figure 2:**
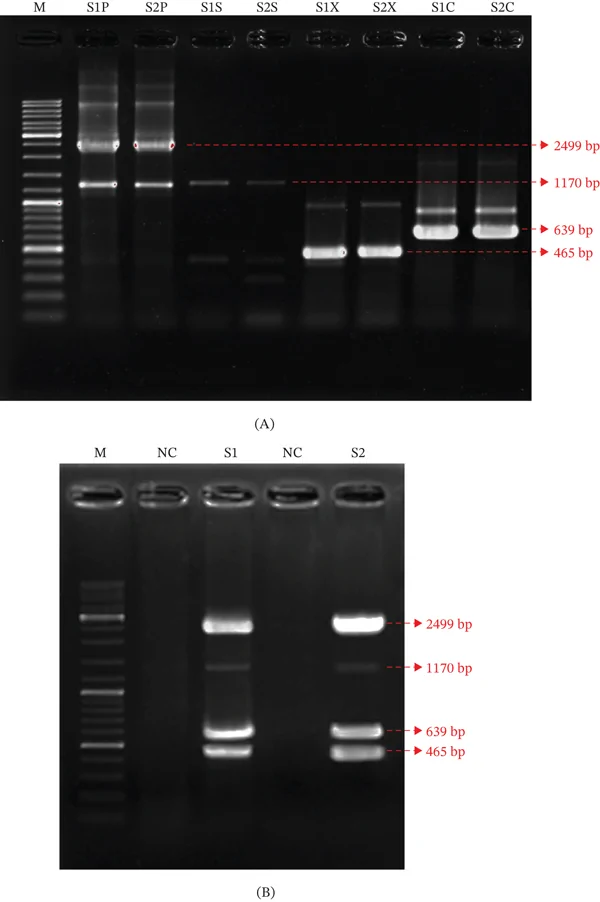
PCR amplification and purification of HBV overlapping ORFs. (A) PCR amplification of the four ORFs of the HBV genomes, resolved on a 1.5% agarose gel. (B) Purified products of the complete ORFs, pooled together and visualized on a 1.5% agarose gel. Distinct bands were observed at the expected sizes: polymerase (P), 2499 bp; surface (S), 1170 bp; core (C), 639 bp; and X, 465 bp. M, DNA marker; S1 and S2, samples 1 and 2; P, polymerase ORF; S, S ORF; X, X ORF; C, C ORF; NC, negative control.

The HBV genome consists of partially double‐stranded DNA that can be amplified using multiple primer sets. In this study, we sequenced the complete HBV genomes using an improved amplification‐based sequencing strategy. Conventional PCR, performed with four pairs of primers targeting the complete ORFs, successfully amplified four overlapping fragments of the expected sizes: 2499 bp (P gene), 1170 bp (S gene), 639 bp (C gene), and 465 bp (X gene) (Figure 2A). Agarose gel electrophoresis confirmed the specific amplification of these fragments, with no nonspecific DNA bands observed (Figure 2B). The four purified ORFs from each HBV genome were pooled and subjected to Illumina sequencing. High‐throughput sequencing generated a total of 22.4 million raw reads. After quality trimming and adapter removal, reads longer than 50 bp were retained and mapped (~0.94 million reads) to the HBV reference genome, resulting in complete (100%) genome coverage. The assembled viral genome exhibited an exceptionally high mean sequencing depth of 563,787x. The assembled genomes of HBV associated with OBI in MHD patients were 3182 bp in length, with GC contents of 48.7% and 48.8%, respectively. BLAST analysis and sequence alignment with reference genomes (GenBank accession No. MF925358.1) confirmed the completeness of the assembled sequences. Each genome contained four majors overlapping ORFs: P (2499 bp), S (1170 bp), C (639 bp), and X (465 bp). The complete genome sequences of two HBV, MGH_HBV‐076 and MGH_HBV‐104, have been deposited in GenBank under accession numbers PV982281 and PV982280, respectively.

### 3.5. Phylogenetic Classification and Genotyping of HBV

**Figure 3:**
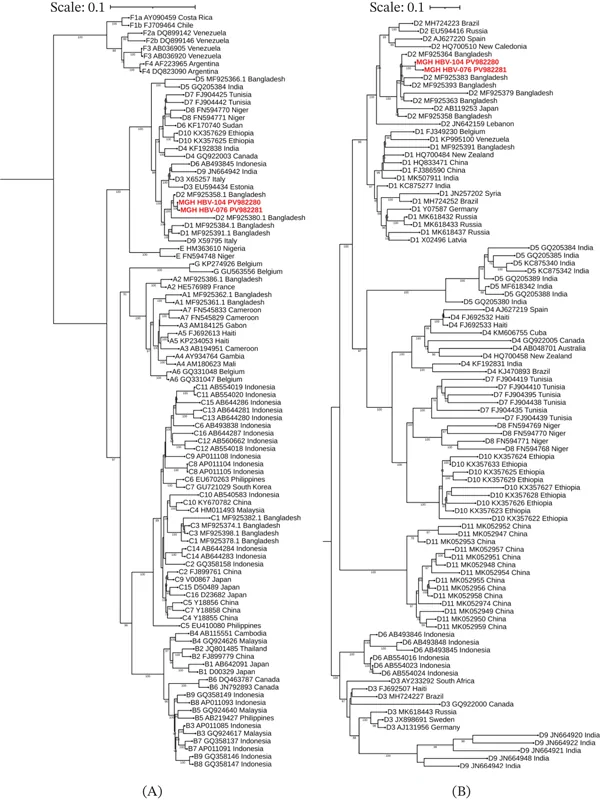
Phylogenetic analysis and genotyping of complete HBV genome sequences. A maximum likelihood phylogenetic tree was constructed using IQ‐TREE2 with 1000 bootstrap replicates under the GTR + F + I + G4 model. Bootstrap values ≥ 70% are shown at the corresponding nodes. Two newly sequenced HBV from this study are highlighted in red to indicate their phylogenetic placement relative to global HBV genomes. (A) Phylogeny of 99 complete genomes representing Genotypes A–G and their subgenotypes. (B) Phylogeny of 98 complete genomes restricted to Genotype D and its subgenotypes.

Phylogenetic analysis using 97 reference sequences representing different HBV genotypes and subgenotypes demonstrated that the HBV from this study (MGH_HBV‐076 and MGH_HBV‐104) clustered within Genotype D (Figure 3A). Both HBV genomes (PV982281 and PV982280) showed a close evolutionary relationship with previously reported HBV genomes from Bangladesh, indicating a shared lineage. Moreover, analysis of 98 complete genomes representing various subgenotypes of Genotype D revealed that the HBV genomes clustered specifically within Subgenotype D2 (Figure 3B). This finding was further validated by genotyping with the well‐established online server geno2pheno, confirming that both HBV genomes belong to Subgenotype D2 under Genotype D.

### 3.6. Genome‐Wide Mutational Profile of HBV

**Figure 4:**
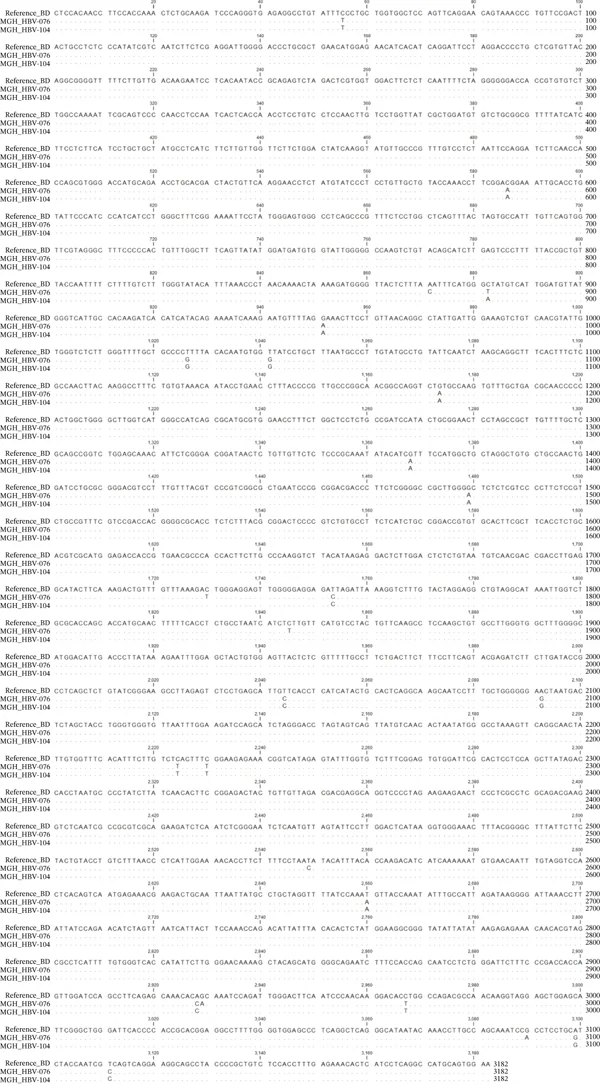
Nucleotide mutational analysis of complete HBV genomes. Mutations were identified by aligning the full‐length HBV genome sequences of the studied sequences (MGH_HBV‐076 and MGH_HBV‐104) against the reference genome (MF925358.1). Dots indicate nucleotide residues identical to the reference.

**Figure 5:**
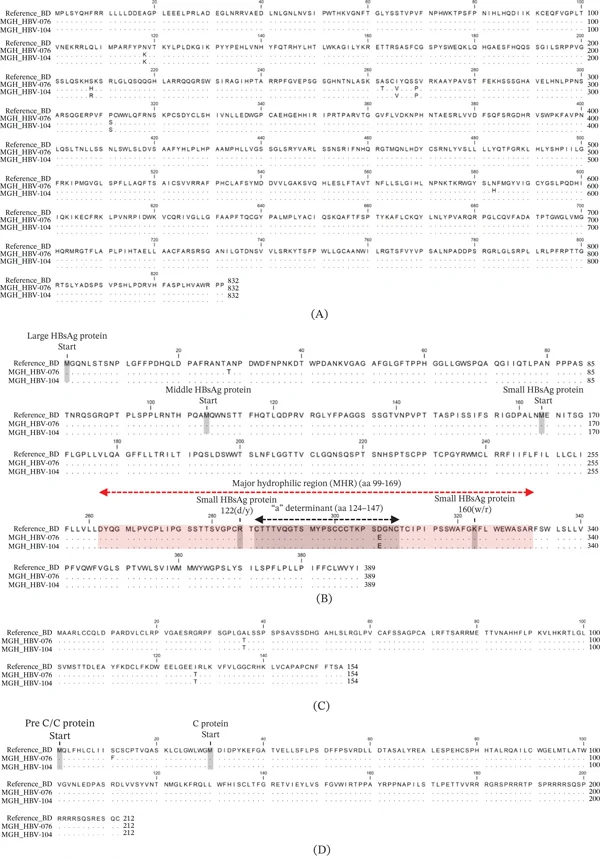
Aa mutational analysis of HBV ORFs. Aa variations of the various proteins of studied HBV genomes were analyzed against reference genome (MF925358.1). (A) Polymerase. (B) Large HBsAg. (C) X protein. (D) PreC/C protein. Dots indicate aa residues identical to the reference sequence.

Comparative analysis of the complete genome sequences of the HBV genomes identified in this study (MGH_HBV‐076 and MGH_HBV‐104) revealed multiple nucleotide variations relative to the reference sequence (MF925358.1) (Table 5, Figure 4). Clinically relevant nucleotide substitutions were detected in both sequences, including T1753C in the basal core promoter (BCP), C1845T in the preC region, G1479A and T1753C in the X region, and C586A in the preS1/preS2/S region (Table 5). In addition, nonsynonymous substitutions (T2660A, A2928C, G2929A, G3090A, A3099G, T3111C, C55T, and A871C) were observed in the ORF P, although their effects remain unknown (Table 5). Aa analysis of MGH_HBV‐104 revealed polymerase mutations S208H/R, A262T, I265V, S269P, and P311S, whereas MGH_HBV‐076 carried an additional N118K mutation (Table 5, Figure 5A). None of these mutations have been previously potentially associated with drug resistance. In the large HBsAg protein, two aa substitutions (A28T, D307E) were identified in MGH_HBV‐076, whereas only one (D307E) was present in MGH_HBV‐104. Notably, the large HBsAg mutation D307E corresponds to D144E in the small HBsAg, located within the MHR and the “a” determinant (Figure 5B). The HBx protein of both HBV genomes carried two mutations (A36T and I127T) (Figure 5C). In the preC region, only MGH_HBV‐076 showed a single aa substitution (S11F), whereas MGH_HBV‐104 showed no detectable changes compared with the reference sequence (Figure 5D). Furthermore, mutational analysis using the geno2pheno tool revealed multiple aa substitutions in the reverse transcriptase (RT) domain of the polymerase protein (rtN53D, rtY54H, and rtH126R) in both HBV genomes, none of which are linked to drug resistance [44]. However, both sequences carried HBsAg escape mutations (A128V and D144E).

**Table 5:** Mutational analysis of HBV genome of four overlapping ORF and their potential effects.

Region	Mutation (nt) in complete genome	Mutation (aa) in ORF	Possible effect	References
PreC–C region				
PreC (nt1814‐1900)	C1845T	S11F	EN‐CHB	[35]
Core (nt1901‐2452)	T2044C	Synonymous	EU	[36]
	A2092G	Synonymous	EU	In this study
	C2224T	Synonymous	EU	In this study
	C2230T	Synonymous	EU	In this study

X region				
X (nt1374‐1838)	G1479A	A36T	CHB	[37]
	C1730T	Synonymous	EU	In this study
BCP (nt1742‐1849)	T1753C	I127T	CHB/HCC	([38]; [39])

PreS1/preS2/S region				
PreS1(nt2848‐3171)	A2928C	Synonymous	EU	In this study
	G2929A	A28T	EU	In this study
	C2967T	Synonymous	EU	In this study
	G3090A	Synonymous	EU	In this study
	A3099G	Synonymous	EU	In this study
	T3111C	Synonymous	EU	In this study
PreS2 (nt3172‐154)	C55T	Synonymous	EU	In this study
S (nt155‐835)	C586A	D144E	Immune/vaccine escape mutation	([40]; [41])

Polymerase gene region				
TP domain (nt2307‐2837)	T2549C	Synonymous	EU	In this study
	T2660A	N118K	EU	In this study
Spacer domain (nt2838‐129)	A2928C	S208R	EU	In this study
	G2929A	S208H	EU	In this study
	C2967T	Synonymous	EU	In this study
	G3090A	A262T	EU	In this study
	A3099G	I265V	EU	In this study
	T3111C	S269P	Genotyping	[42]
	C55T	P311S	EU	In this study
RT domain (nt130‐1161)	C586A	Synonymous	EU	In this study
	A871C	N583H	EU	[43]
	C882T	Synonymous	EU	In this study
	G951A	Synonymous	EU	In this study
	T1026G	Synonymous	EU	In this study
	T1041G	Synonymous	EU	In this study
RNaseH domain (nt1162‐1620)	G1173A	Synonymous	EU	In this study
	G1368A	Synonymous	EU	In this study
	G1479A	Synonymous	EU	In this study

Abbreviations: CHB, chronic Hepatitis B; EU, effect unknown; EN‐CHB; Hepatitis B e antigen‐negative; HCC, hepatocellular carcinoma.

### 3.7. Impact of Aa Changes on HBsAg Antigenicity

**Figure 6:**
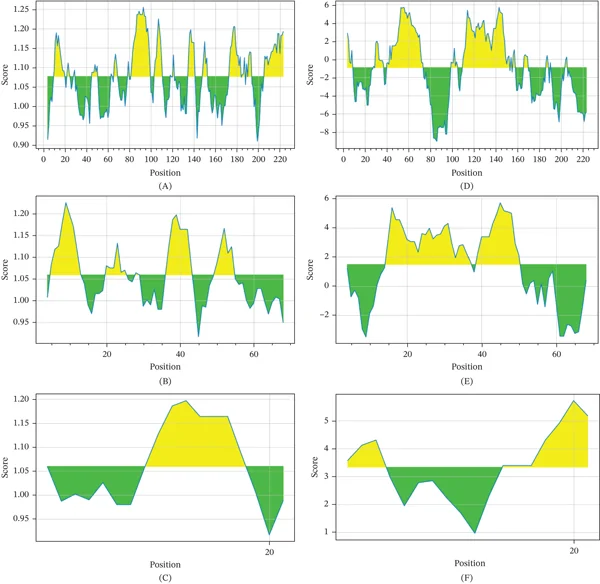
Antigenicity and hydrophilicity profiles of HBsAg in the identified HBV. Antigenicity and hydrophilicity plots were generated using the Immune Epitope Database (IEDB) analysis resource to predict antigenic and hydrophilic properties. (A) Antigenicity of full‐length HBsAg. (B) Antigenicity of the major hydrophilic region. (C) Antigenicity of the “a” determinant. (D) Hydrophilicity of full‐length HBsAg. (E) Hydrophilicity of the major hydrophilic region. (F) Hydrophilicity of the “a” determinant. Scores above the threshold line (yellow areas) indicate predicted antigenic or hydrophilic regions, whereas scores below the threshold line (green areas) indicate nonantigenic or hydrophobic regions.

Aa substitutions within the MHR, particularly in the “a” determinant, are known to influence the antigenicity and immunogenicity of HBsAg. Antigenicity analysis of the HBsAg sequences revealed only minor variations compared with the reference sequences, with no significant reduction in antigenicity (Table 6, Figure 6A). The MHR, including the “a” determinant, consistently showed high antigenicity scores (Figure 6B,C). Additionally, hydrophilicity profiles generated using IEDB also showed broadly comparable patterns of hydrophilic and hydrophobic regions among the analyzed sequences (Table 6, Figure 6D–F).

**Table 6:** Antigenicity and hydrophilicity profile.

Region	Threshold	Minimum	Maximum
	This study (ref)	This study (ref)	This study (ref)
Antigenicity properties			
HBsAg	1.076 (1.076)	0.909 (0.909)	1.255 (1.255)
Major hydrophilic region	1.059 (1.059)	0.917 (0.919)	1.225 (1.225)
“a” determinant	1.060 (1.061)	0.917 (0.919)	1.197 (1.197)
Hydrophilic properties			
HBsAg	−0.919 (−0.909)	−9.029 (−9.029)	5.714 (6.029)
Major hydrophilic region	1.482 (1.516)	−3.500 (−3.500)	5.714 (6.029)
“a” determinant	3.329 (3.399)	0.957 (0.957)	5.714 (6.029)

*Note:* IEDB was used to perform the analysis of antigenicity and hydrophilicity under the Kolaskar and Tongaonkar antigenicity and Parker hydrophilicity prediction parameter, respectively. HBsAg, major hydrophilic region, and “a” determinant protein region were analyzed for both HBV genomes. These regions were identical for both HBV genomes showing similar antigenic and hydrophobic scores. Values inside the brackets indicate the antigenicity and hydrophilicity of reference sequence.

## 4. Discussion

The prevalence of OBI among HD patients varies widely, ranging from 0% to 58%, largely depending on regional HBV endemicity [17, 45, 46]. This study is aimed at establishing a modified workflow for complete HBV genome sequencing by integrating conventional PCR amplification with Illumina sequencing technology. Additionally, we performed comparative genomic characterization and mutational analysis of two HBV genomes identified from MHD patients with OBI.

Age and sex are important factors in MHD, with older adults and male populations predominating because of rapid CKD progression and intrinsic biological differences [47–51]. Similar findings were observed in this study, where the majority of patients were older and male; most were retired persons, farmers, or housewives. This profile is commonly associated with restricted access to healthcare services, concerns about the disease, and limited financial means [50]. Additionally, in this study, diabetes mellitus and hypertension were more frequently observed in men, and these are reported as leading causes of CKD, consistent with previous studies [50, 52]. In contrast to the global standard of thrice‐weekly HD, the majority of patients in this study received dialysis twice weekly, which may have adversely affected clinical outcomes [53, 54]. Notably, most of the patients in this study were fully unvaccinated, reflecting the persistently low vaccination coverage commonly observed in resource‐limited settings [55, 56]. A very small proportion of patients demonstrated anti‐HBc positivity, suggesting prior exposure to the Hepatitis B virus, whereas the majority exhibited anti‐HBs titers at protective levels. These results may vary across regions, as demonstrated by studies from Egypt, Saudi Arabia, Iran, and Korea [57–60].

Classical OBI is characterized by the presence of low HBV DNA in peripheral blood/hepatic tissue [46, 61]. Besides, several previous studies reported that false‐negative results in commercial ELISA assays though the patients were with high viral load, which might be because of diagnostic escape variants and mutations affecting HBsAg antigenicity (Atypical OBI) [14, 27, 62–64]. Accordingly, two OBI cases identified in this study showed high viral loads, which might termed as atypical OBI and consistent with virological mechanisms [7, 14, 27, 62–65]. The OBI prevalence among MHD patients in this study was 3.5%, which is higher than that reported in the general population of Bangladesh (1.8%), aligning with previous reports from Iran, Italy, and India [66–69].

The HBV genome is a partially double‐stranded, circular DNA molecule that completes its structure during intracellular replication [70]. Traditionally, complete genome sequencing has been achieved through amplification of overlapping fragments using PCR followed by Sanger sequencing or by next‐generation sequencing [34, 43, 71]. In this study, we successfully sequenced the complete genomes of two HBV by amplifying four ORFs with PCR followed by Illumina sequencing, and the results were consistent with known genomic features [72–74].

Genotype D has been reported to increase the risk of chronic infection, progression to cirrhosis, and HCC compared with other genotypes [75, 76]. Phylogenetic analysis in this study confirmed that both HBV genomes belonged to Genotype D (Subgenotype D2) and the ayw3 serotype [77]. The observed phylogenetic clustering suggests the preliminary signal of this subgenotype in Bangladesh, underlining the importance of molecular surveillance to prevent nosocomial transmission. Anti‐HBc positivity is a strong risk marker for OBI [78, 79]. Both patients in this study tested positive for total anti‐HBc and anti‐HBs, though their anti‐HBs titers were below protective levels. Previous studies have reported that immune dysfunction associated with CKD and long‐term dialysis may facilitate viral persistence [7, 13]. Notably, the two patients in this study were undergoing HD twice and once weekly, respectively, which may have contributed to viral persistence and increased risk of OBI [13].

Genetic variations within the preS1 region can affect the antigenicity of large HBsAg and have been implicated in HCC development [27, 80]. In this study, one novel so far nonsynonymous mutation (G2929A, resulting in A28T substitution) was identified in preS1, which has not been reported previously. Additionally, mutations within the “a” determinant (aa 124‐147) of the S gene are known to alter antigenic conformation and HBsAg secretion, leading to false‐negative results in commercial assays [10–13]. Both HBV genomes in this study carried a point (D144E) mutation in the “a” determinant, which may contribute to diagnostic, vaccine/immune escape, and is frequently associated with OBI [41, 81–84]. The detection of an A128V substitution in HBsAg supports the potential for diagnostic escape, as similar mutations have previously been linked to reduced assay sensitivity and vaccine escape [82]. However, antigenicity analysis compared with reference sequence showed no significant differences in predicted antigenicity or hydrophilicity (threshold values: 1.060 and 1.061), likely because of the limited number of aa variations [14, 27].

Mutations in the BCP and preC regions can suppress or abolish HBeAg expression, allowing viral evasion of host immune responses and increasing the risk of progressive liver disease [43, 85]. Nonsynonymous substitution C1845T in the preC region is potentially associated with HBe antigen‐negative CHB infections [35]. Similar mutations were observed in this study. Variations in the X region, encoding the HBx protein, also contribute to hepatocyte injury and tumorigenesis [86]. The aa substitutions identified in the BCP, X, and preC regions in this study are likely associated with chronic Hepatitis B (CHB) and HCC, as reported in earlier studies [35, 36, 38, 39]. Notably, the T1753C and G1479A substitutions in the BCP and X regions, respectively, have been potentially associated with CHB and HCC [36–39].

Mutations in the RT domain are known to drive antiviral resistance and are critical for HBV polymerase function [43]. In this study, one nonsynonymous mutation was identified in the RT domain, previously reported but with unknown functional consequences [43]. Importantly, no established drug resistance mutations were detected. Additional nonsynonymous mutations were observed in the spacer domain (six mutations) and terminal protein (TP) domain (one mutation). However, the significance of the identified mutations remains unclear, except for T3111C in the spacer domain, which results in an S269P substitution and associated with viral genotyping [42].

## 5. Conclusion

The findings of the current study demonstrate a modified approach for complete HBV genome sequencing, which may be valuable for large‐scale genomic surveillance. Both HBV genomes (PV982281 and PV982280) were classified as Subgenotype D2 and Subtype ayw3 and carried key mutations in preS1, the “a” determinant of the S gene, BCP, preC, X, and polymerase regions. These mutations may contribute to immune escape, reduced HBsAg detectability, and increased HCC risk in HD patients. Collectively, these preliminary results highlight the need for genomic surveillance of OBI among HD patients to inform effective prevention and control strategies for HBV infection.

## Author Contributions

M.G.H. conceived the study. R.M., P.I.D., N.S.I., and C.D. collected the samples. R.M., A.M., P.I.D., S.M.N.H., and A.D. conducted all kinds of experiments. R.M., M.S.R., and A.M. analyzed the data. R.M. and M.G.H. wrote the draft of the manuscript. S.A., S.M.R.U.I., S.S., T.I., M.U.A., C.R.D., and M.G.H. reviewed, edited, and finalized the manuscript.

## Funding

This study was funded by the Medical Education and Family Welfare Division, Ministry of Health and Family Welfare, People′s Republic of Bangladesh.

## Disclosure

All authors have read and agreed to the publish manuscript.

## Conflicts of Interest

The authors declare no conflicts of interest.

## Data Availability

The data that support the findings of this study are available from the corresponding author upon reasonable request.
